# Prevalence of Aminoglycoside Resistance Genes in Clinical Isolates of *Pseudomonas aeruginosa* from Taif, Saudi Arabia—An Emergence Indicative Study

**DOI:** 10.3390/microorganisms11092293

**Published:** 2023-09-12

**Authors:** Shaymaa W. El-Far, Mohamed W. Abukhatwah

**Affiliations:** 1 Division of Pharmaceutical Microbiology, Department of Pharmaceutics and Industrial Pharmacy, College of Pharmacy, Taif University, Taif 21974, Saudi Arabia; 2 Pediatric Nephrology Section of Pediatric Department, Alhada Armed Forces Hospital, Taif 26792, Saudi Arabia; abukhatwah@hotmail.com

**Keywords:** *P. aeruginosa*, AMEs, *aac*(*6*′)-*Ib*, *ant*(*3*″)-*I* and *aph*(*3*′)-*VI*

## Abstract

Hospital-acquired infections caused by *P. aeruginosa* contribute to global distress because of the elevated rates of microbial antibiotic resistance. Aminoglycosides are antipseudomonal agents that are effectively and frequently utilized to eradicate this infection. This current study is a retrospective study investigating plasmid-mediated aminoglycoside resistance by focusing on the prevalence of the genes encoding aminoglycoside-modifying enzymes (AMEs) and 16S rRNA methylase among *P. aeruginosa* clinical isolates from Taif, Saudi Arabia. A hundred clinical isolates of *P. aeruginosa* were collected. The isolates were identified from February 2021 to February 2022. Antibiotic susceptibility testing and MICs were determined using (DD) and (BM-MIC) testing, respectively. AMEs and 16S rRNA methylase variants in bacterial isolates were amplified via PCR for genetic detection. A relatively high multiple antibiotic resistance rate corresponding to 10–32% was reported. Eighteen percent of *P. aeruginosa* isolates were gentamicin–amikacin–tobramycin resistant according to the MIC levels. The aminoglycoside-resistant strains were additionally identified via *GyrA* gene sequencing. The phylogenic relatedness dendrogram of the sequenced *GyrA* genes was performed using a neighbor-joining method via MEGAX software version 10.2.6. The most prevalent AME encoding gene was *aac*(*6*′)-*Ib*, observed in 94.4% of resistant isolates, while a resistance gene cocktail of [*aac*(*6*′)-*Ib* and *ant*(*3*″)-*I*] was a highly frequent combination (27.8%). This study updated the knowledge about aminoglycoside resistance mechanisms in *P. aeruginosa*, which constitutes an urgent need, especially after the COVID-19 crisis, which was associated with increased antimicrobial use and resistance rates.

## 1. Introduction

*P. aeruginosa* is one of the major opportunistic pathogens that cause acute or chronic infections. On the parallel side, illogical and widespread use of antibiotics has led to long-term impact on the antimicrobial resistance (AMR) of such microorganisms [1,2,3].

Aminoglycosides are a large category of antibiotics that bind specifically to 16S rRNA in 30S ribosomal subunits and disturb protein translation. They are extensively used in the treatment of serious bacterial infections, especially aerobic Gram-negative bacteria. The increasing problem of multi-resistance in Gram-negative bacteria, such as *P. aeruginosa,* warrants new studies focused on understanding aminoglycoside resistance [4]. The widespread occurrence of antibiotic-resistant strains of *P. aeruginosa* in hospitals is a matter of growing resistance as they cause various types of hospital-acquired human infections, with an expected elevation of resistance rates due to the antibiotic-dependent protocols that had already been utilized during the COVID-19 pandemic [5].

ARGs acquisition represents the major cause of plasmid-mediated aminoglycoside resistance via the encoding of AMEs, namely acetyltransferases (*aac*), phosphotransferases (*aph*), and nucleotidyl transferases (*ant*) [6]. In addition, 16S rRNA methylases (*rmts*) are another aminoglycoside resistance pathway among clinical *P. aeruginosa* isolates [7]. The *aac*(*6*′)-*Ib* gene has an obvious relationship to high gentamicin resistance; it is found in the majority of Gram-negative bacteria, including *P. aeruginosa* [7,8]. Other common AME encoded by *ant*(*3*″)-*I*, *aph*(*3*′)-*VI*, *ArmA*, *aac*(*3*′)-*II*, and *aac*(*6*′)-*II* was investigated in pan-resistant *P. aeruginosa* [9,10].

The resistance mechanism complexity was encountered by the co-existence of more than one ARG, where the spread of these genes is particularly based on the type of bacterium causing the infection and misuse of aminoglycosides among different hospitals or geographic regions [11]. This current study is a retrospective, observational analytical study aimed at detecting the antibiotic resistance patterns of *P. aeruginosa* clinical isolates and focusing on the mechanism of resistance against the frequently used aminoglycosides (gentamicin, amikacin, and tobramycin) and the prevalence of the genes encoding for resistance in *P. aeruginosa* clinical isolates. Furthermore, it displays a general view of the probability of resistant microbial transmission.

## 2. Materials and Methods

### 2.1. Collection of P. aeruginosa Isolates

We recovered 100 clinical isolates as a microbiology laboratory procedure from the microbiology laboratory at King Abdulaziz Specialist Hospital from February 2021 to February 2022, and all isolates were obtained from adult male/female patients (above 18 years old). Children and pregnant women were not included in this study. All strains were initially recovered on MacConkey’s agar (Oxoid, Basingstoke, UK) and then purified on cetrimide agar (Scharlau, Barcelona, Spain). All isolates were primarily identified by the Vitek 2^®^ system (BioMérieux, Craponne, France) and API 20NE^®^ (BioMérieux, Craponne, France). The genus level was also confirmed via the amplification of the *algD* gene using the primer pairs (Macrogen, Geumcheon-gu, Seoul, Republic of Korea) listed in Table 1. Long-term storage of isolates at −84 °C in glycerol brain/heart infusion was undertaken for further tests. This study was performed with ethical approval No. 42-0107 following the regulations of the ethical committee at Taif University.

### 2.2. Antimicrobial Susceptibility Testing (AST)

Both DD and BM-MIC testing was conducted among all isolates using Muller Hinton agar and broth, respectively [12]. Breakpoints for different antibiotics were interpreted based on the guidelines of CLSI, 2017 [13]. Seven antibiotics that represent different categories of antimicrobial agents were utilized, including three aminoglycosides (gentamicin—10 µg (GM), amikacin—30 µg (AK), and tobramycin—10 µg (TM)) and ceftazidime—30 µg (CTZ), imipenem—10 µg (IMP), piperacillin/tazobactam—100/10 µg (TZP), and ciprofloxacin—5 µg (CIP) (Merseyside, United Kingdom). MICs were determined for the mentioned 7 antibiotics utilizing a wide concentration range from (0.5–1024 µg/mL), as shown in Table 2.

**Table 1:** Primer sets and PCR cycling conditions were used for genotyping and amplification of aminoglycosides resistance genes.

Primer/Gene	Sequence	PCR Condition	Amplicon Size (pb)	References
*VIC*/*algD*	F: TTCCCTCGCAGAGAAAACATC R: CCTGGTTGATCAGGTCGATCT	Initial denaturation at 95 °C for 15 min, then 30 cycles of 95 °C for 1 min, 58 °C for 1 min and 72 °C for 5 min and one cycle of final elongation at 72 °C	520	[14]
*GyrA*	F: TTATGCCATGAGCGAGCTGGGCAACGACT R: AACCGTTGACCAGCAGGTTGGGAATCTT	Initial denaturation at 95 °C for 15 min, then 35 cycles of 95 °C for 1 min, 57 °C for 1 min, and 72 °C for 5 min, and one cycle of final elongation at 72 °C.	365	[2]
*Aph*(*3*′)-*VI*	F: ATGGAATTGCCCAATATTATT R: TCAATTCAATTCATCAAGTTT	Initial denaturation at 95 °C for 15 min, then 30 cycles of 95 °C for 1 min, 55 °C for 1 min, and 72 °C for 5 min, and one cycle of final elongation at 72 °C.	780	[15]
*aac*(*3*′)-*II*/*aac*(*3*′)-*II*	F: ATATCGCGATGCATACGCGG R: GACGGCCTCTAACCGGAAGG		877	
*aac*(*6*′)-*Ib*/*aac*(*6*′)-*Ib*	F: TTGCGATGCTCTATGAGTGGCTA R: CTCGAATGCCTGGCGTGTTT		472	
*aac*(*6*′)-*II*/*aac*(*6*′)-*II*	F: CGACCATTTCATGTCC R: GAAGGCTTGTCGTGTTT		542	
*ant*(*3*″)-*I*/*ant*(*3*″)-*I/*	F: CATCATGAGGGAAGCGGTG R: GACTACCTTGGTGATCTCG		787	
*ArmA*/*ArmA*	F: CCGAAATGACAGTTCCTATC R: GAAAATGAGTGCCTTGGAGG		846	
*rmtB*/*rmtB*	F: ATGAACATCAACGATGCCCTC R: CCTTCTGATTGGCTTATCCA	Initial denaturation at 95 °C for 15 min, then 30 cycles of 95 °C for 1min, 60 °C for 1 min, and 72 °C for 5 min, and one cycle of final elongation at 72 °C.	769	[15]

### 2.3. Genomic DNA Extraction

Genomic DNA was extracted using Gene JET Genomic DNA Purification Kit (Thermo Fisher Scientific, Waltham, WA, USA) according to the manufacturer’s protocol.

### 2.4. PCR and Gel Electrophoresis

Genetically identification of Pseudomonal isolates was investigated via *algD* PCR amplification (Macrogen, Geumcheon-gu, Seoul, Republic of Korea). Furthermore, GyrA Sequencing was performed on aminoglycosides resistant isolates only as an extra confirmatory tool.

ARGs PCRs were carried out on all isolates. Primers, cycling conditions, and amplicon sizes are listed in Table 1 (references in Table 1). Amplification of targeted ARGs include (*Aph*(*3*′)-*VI*, *aac*(*3*′)-*II*, *aac*(*6*′)-*Ib*, *aac*(*6*′)-*II*, *ant*(*3*″)-*I*, *Arm*, and *rmtB*). The target genes were amplified in the total PCR reaction mixture of 20 µL containing (4 µL of DNA, 4 µL of 5× master mix (Solis BioDyne, Tartu, Estonia), 0.6 µL for each of forward and reverse primer (10 pmol/μL)), *P. aeruginosa ATCC 27853* was utilized as a reference. Visualization PCR fragments (2 µL of RCR product) carried out on 1.5% agarose gels with 0.3 µg/mL ethidium bromide (EtBr) and GeneRuler 100 bp DNA Ladder, using a power supply (Labnet International Inc., Taipei, Taiwan).

### 2.5. DNA Sequencing

The purified PCR products of the *GyrA* gene were purified, and Sanger sequenced in one direction by capillary electrophoresis sequencing (CES) utilizing the forward primer. Nucleotide sequences were determined at the Macrogen sequencing facility (Macrogen Inc., Seoul, Republic of Korea).

*GyrA* gene sequences were corrected using the molecular evolutionary genetic analysis MEGAX software version 10.2.6, (Bio Design Institute, Tempe, AZ, USA). Homology searches of nucleotide sequences were performed via FASTA and BLAST screen. Original nucleotide sequences of *GyrA* were obtained from the GenBank nucleotide sequence database with accession numbers L29147 [2].

Molecular phylogenetic analysis of the sequenced *GyrA* genes of resistant pseudomonal isolates was performed using the neighbor-joining method via MEGAX version 10.2.6.

## 3. Results

All the isolates under investigation in this study were unduplicated and obtained from different body parts at both male and female wards at the tertiary health care center (King Abdulaziz Specialist Hospital) in Taif City, Saudi Arabia, including sputum (n = 44), urine (n = 36), blood (n = 7), wound swap (n = 7), bile (n = 2), and (n = 1) for each of eye swab, vaginal swab, peritoneal fluid, and catheter tip, as shown in Table S1.

The resistance profiles of the *P. aeruginosa* isolates were determined using both DD and BM-MIC methods. Table 2 summarizes all the resulting data obtained from these methods. A paired *t*-test was conducted for the differences between the two methods in the means of percentage of resistant/susceptible isolates (% R:S) and [total number of resistant and intermediate isolates/susceptible isolates [% (R + I):S]. The statistical analysis and data interpretations revealed a strong correlation between the two methods with a confidence level of 0.95, as presented in Table S2.

According to BM-MIC, the tested isolates showed that 32.0% (32/100) were resistant to one or more antibiotics as 31.0% (31/100) were resistant to gentamicin, 18.0% (18/100) to both amikacin and tobramycin, 10.0% (10/100) to ceftazidime, 32.0% (32/100) to imipenem, 13.0% (13/100) to piperacillin/tazobactam, and 29.0% (29/100) to ciprofloxacin, as presented in Table 2. The corrected *GyrA* sequences were uploaded to the National Center for Biotechnology Information (NCBI), Accession no. (OR188196 to OR188213). The aminoglycoside-resistant isolates were subjected to homology searches of their *GyrA* sequences. The results indicated a high identity percentage of 99.32–99.67% compared to the reference nucleotide sequence of *GyrA* obtained from the GenBank nucleotide sequence database (accession number L29147). This finding confirms the previous genetic identification of the isolates (Figure S1).

Among all isolates, twenty-nine (29%) strains were gentamicin-resistant via DD in contrast to (31%) using the BM-MIC method. However, both methods showed the same amikacin and tobramycin-resistant rate (18%). Generally, eighteen isolates were resistant to all the aminoglycosides assayed (gentamicin, amikacin, and tobramycin). It is important to note that the MIC of piperacillin-tazobactam was determined using a constant concentration of tazobactam (4 µg/mL) [16].

For the same antibiotic, elevated MIC was observed compared to reduced inhibition zone diameter and vice versa due to the concurrent existence of a wide range of resistance rates from high, intermediate, and low-resistant strains [17].

All ARGs focused in this study (*aac*(*6*′)-*Ib*, *ant*(*3*″)-*I*, *aph*(*3*′)-*VI, armA*, *aac* (*3*′)-*II*, *aac*(*6*′)-*II* and *rmtB* were amplified via PCR according to the conditions and primer sets mentioned previously. The resulting PCR products were (472 bp for *aac*(*6*′)-*Ib*, 780 bp for *aph*(*3*′)-*VI*, 787 bp for *ant*(*3*″)-*I*, 846 pb for *armA,* 877 bp for *aac*(*3*′)-*II*) while *rmtB* was completely absent in all isolates under investigation. The PCR fragments were separated on 1.5% agarose gel using a 100-base pair DNA Ladder. The resistance profile of pan aminoglycoside isolates is reported in Table 3.

**Figure 1:**
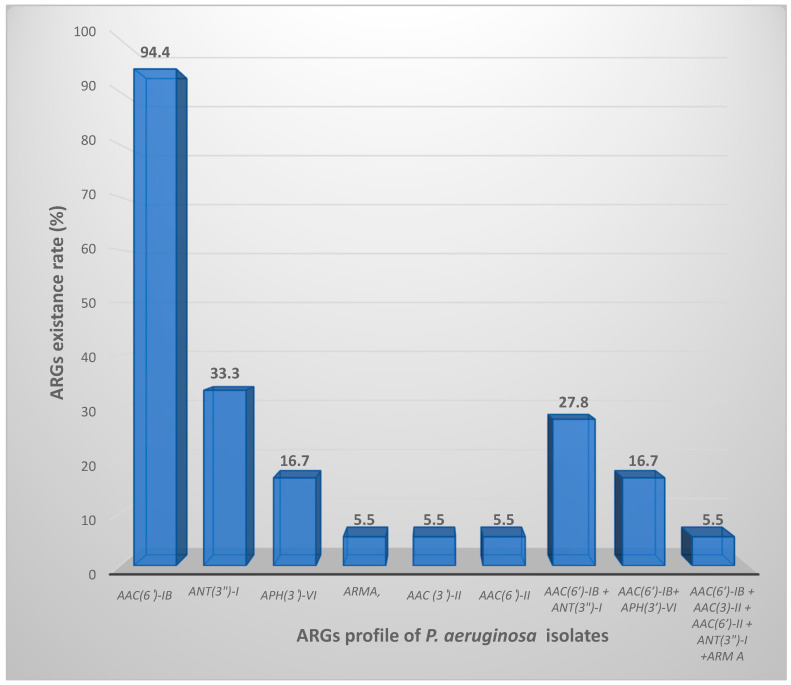
ARGs distribution profile among the isolated *P. aeruginosa* resistant strains.

The most frequent ARGs was *aac*(*6*′)-*Ib*, detected in 94.4% (17/18) of the resistant strains, followed by *ant*(*3*″)-*I* (6/18, 33.3%), then *aph*(*3*′)-*VI* (3/18, 16.7%), while *armA*, *aac*(*3*′)-*II* and *aac*(*6*′)-*II* were found in (1/18, 5.5%). Conversely, *rmtB* was negative in all the isolates under investigation (Figure 1).

Nine isolates were investigated with more than one ARG. The genetic combination of *aac*(*6*′)-*Ib* and *ant*(*3*″)-*I* was the highly frequent one (5/18, 27.8%) followed by *aac*(*6*′)-*Ib* and *aph*(*3*′)-*VI* (3/18, 16.7%). One isolate harbored five genes: *aac*(*6*′)-*Ib + aac* (*3*′)-*II + aac*(*6*′)-*II + ant*(*3*″)-*I + arm A.* Although resistance phenotypes against gentamicin and/or amikacin were observed, negative tests for ARGs had been evaluated in this study. As expected, all the susceptible isolates were free from ARGs under the test. The findings of this study revealed that the most common ARG was *aac*(*6*′)-*Ib* corresponding to (17/18, 94.4%)*,* followed by *ant*(*3*″)-*I* (6/18, 33.3%), *aph*(*3*′)-*VI* (3/18, 16.7%). Furthermore, the detection rate for each *armA*, *aac*(*3*′)-*II*, and *aac*(*6*′)-*II* was equal occurring in 1 out of 18 isolates (5.5%). Conversely, *rmtB* was negative in any of the isolates. Although resistance phenotypes against gentamicin and/or amikacin were observed, negative tests for ARGs had been evaluated in this study. As expected, all the susceptible isolates were free from ARGs under the test. All 18 aminoglycoside-resistant strains included in this study were classified as MDR due to their resistance to at least one antibiotic from three different classes: Aminoglycoside, Carbapenems, and Quinolones. The MIC ranges of resistant Pseudomonal isolates were visually represented in Table 1 using red color.

**Figure 2:**
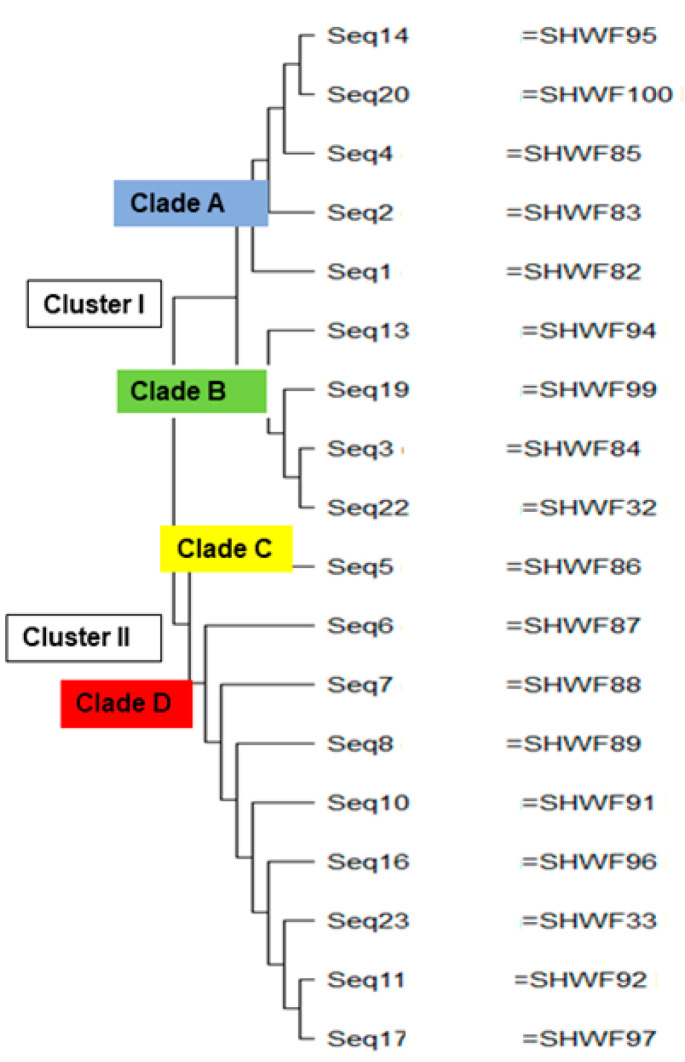
Phylogenetic relatedness between the sequenced *GyrA* genes of aminoglycoside-resistant *P. aeruginosa* using the neighbor-joining method.

Molecular phylogenetic analysis of the sequenced *GyrA* genes using the neighbor-joining method via MEGAX version 10.2.6. revealed four distinct phylogenic clades [clade A, B, C, and D] within two major clusters denoted as Cluster I and Cluster II as depicted in Figure 2. Notably, all the sequences in the clade A [seq14, seq20, seq4, seq2, and seq1] were isolated from sputum specimens in the male ward. Clade B primarily consisted of seq13, seq19, seq3, and seq22 obtained from sputum specimens in the male ward, except for sequence no.19, which was derived from a wound swap in the female ward. In Cluster II, clade C contained a single sequence no. 5 obtained from a wound swap in the female ward. Within clade D, all sequences [seq.6, seq.7, seq.8, seq.10, seq.16, seq. seq.23, seq.11, and seq.17] were from the male ward, except no. 16, which was isolated from a female wound swab.

## 4. Discussion

Despite the ongoing usefulness of aminoglycoside as a group of antipseudomonal agents. The issue of resistance remains a growing concern, with consideration given to geographical variations. Like other antibiotics, there are regional disparities in resistance rates that directly reflect a prescription pattern and the efficacy of infection control measures. [18]. This study exhibited a significant prevalence of plasmid-mediated *P. aeruginosa* resistance with 10–32% of isolates that were resistant among the selected antibiotics [aminoglycosides (gentamicin, amikacin, and tobramycin), piperacillin/Tazobactam, ceftazidime, imipenem, and ciprofloxacin. Although twenty-nine (29%) of isolates were gentamicin-resistant by DD versus (31%) by the BM-MIC method, this result is completely acceptable as there was no statistically significant difference observed between the two methods (DD vs. BM-MIC), Table S2. Our investigation agrees with the study about the evaluation of different gentamicin susceptibility methods among enterobacterial strains. The DD method showed the best compatibility and performance compared to the broth microdilution as a reference method, with a categorical agreement of 98.1% [19].

Our investigation revealed that the rate of aminoglycoside resistance was (18%). This finding in Taif City agrees with another Saudi finding in Hail province, which demonstrated a 16% aminoglycoside resistance rate of *P. aeruginosa* isolates [20,21]. Comparable estimated resistance rate of MDR- *P. aeruginosa* had been reported by NHSN in patients with pneumonia, bacteremia, and UT infections [22]. Recently, highly resistant isolates to antipseudomonal agents have been reported in Qatar [23]. In Egypt, phenotypically and genotypically detection of MDR Gram-negative clinical isolates were reported with rates of gentamicin and amikacin resistance corresponding to (11/26, 42.3%, and 10/26, 38.4%), respectively [24]. Other studies in Kosovo showed an elevated *P. aeruginosa* resistance rate over consecutive 2 years from 2013 to 2015, against each of gentamicin, carbapenems, and ciprofloxacin. Thus, there is a global warrant to emphasize uncontrolled antibiotic use and perfectly adhere to the conception of “reserve drugs” [25].

On the other hand, the findings of a Turkish study reported relatively high gentamicin–mikacin resistance rates of 70.7% and 42.2%, respectively [5]. About twenty years ago, a Saudi study conducted in the Al-Dharan region reported a declining level of MDR—*P. aeruginosa* corresponding to 1–2% regarding the inpatient isolates [26]. Compared to our findings, a warning alarm for a future outbreak of antibiotic resistance should be considered, and regulatory issues and antibiotics stewardship protocols are urgently needed to restrict microbial resistance distribution as soon as possible. Furthermore, there is extreme importance to update our knowledge about the *P. aeruginosa* resistance profiles according to time and geographic regional variations, which ideally give back the diversity of aminoglycoside utilization as antipseudomonal agents, application of the infection quality control and the health care facilities all over the world [27].

In the current work, the highly frequent ARG among resistant isolates was *aac*(*6*′)-*Ib* corresponding to (17/18, 94.4%). This observation was in agreement with an Iranian study that reported 71.2% of the resistant *P. aeruginosa* isolates were *aac*(*6*′)-*Ib* positive [28]. Furthermore, the study investigated the existence of *aph*(*3*′)-*VI* among three *P. aeruginosa* isolates (3/18, 16.7%); these three isolates were found to be highly amikacin resistant with MIC levels between (256–1024 ug/mL), (Table 2). Our finding agreed with *Torres* et al., who reported the contribution of phosphotransferase in amikacin-resistant *P. aeruginosa* strains [29]. Another study in Venezuela investigated that ARGs (*aac*(*6*′)-*Ib*, *aphA1*, and *aad B*) were highly distributed among the examined clinical isolates [30].

In an Iranian study, a mixture of ARGs was demonstrated among tested pseudomonal strains, including [*aac*(*6*′)-*II*, *aad*(*2*″), *and aph*(*3*′)-*VI*], that emphasize the concurrently multiple existences of ARGs in the same isolate. In addition, the work investigated one isolate from 18 that was phenotypically resistant to both gentamicin and amikacin, while it showed a negative genotyping test for ARGs evaluated in this study, this may be revealed to the implication of other variants that were not covered here, as reported elsewhere [31]. In the same manner, a negative test for ARG genotyping was reported for all the susceptible isolates, which agrees with other studies conducted worldwide [32]. An extremely high aminoglycoside resistance was observed previously, combined with a wide range existence of ARGs including different variants of *aad gene* (*A1*, *B*, *A2*), *ant*(*2*″)-*Ia*, *aph*(*3*′)-*IIb*, *aac*(*3*′)-*Ia*, and *aac*(*6*′)-*Iia* [33]. The interplay between efflux pumps *MexXY*, *MexZ,* and the level of *mexXY* expression plays an essential role in aminoglycoside resistance in clinical isolates of *P. aeruginosa*, but the magnitude of the contribution of this efflux pump to resistance is isolate-specific [34].

The molecular phylogenetic analysis of *GyrA* nucleotide sequences reported that almost all the nucleotide sequences isolated from the same hospital ward (the male or female ward) belonged to the same phylogenic group, which indicated a high probability of microbial transmission from colonized or infected patients to other patients or to health care workers and visitors who may subsequently transmit them to others. *Meoli* et al. reported the importance of preventive measures to reduce the prevalence of microbial transmission, especially in the case of surgical site infections [35].

This study highlighted the effect of plasmid-mediated resistance among *P. aeruginosa* as one of the common infection causative agents. Other resistance mechanisms might contribute an axial role in *P. aeruginosa* resistance. Therefore, additional genetic information is required for the implementation of new therapeutic strategies along with infection prevention and quality control policies.

In parallel, this work confirmed the global awareness against microbial multiple drug resistance, including (1) how one microbe harbored more than one resistance mechanism and (2) the phylogenic relatedness analysis reported high expectations of microbial transmission that accelerates the resistance incidence rate So, the current type of studies are obligatory for continuous updating of clinical practice knowledge, to hinder antibiotic resistance, support regulatory aspects and antibiotics stewardship’s programs among aminoglycoside as anti-pseudomonal agents along with other antibiotics.

The limitations of this current study include (1) the local level of data about aminoglycoside resistance mechanisms, (2) limited numbers of ARGs and 16S RNA methylases were searched, especially *rmtA* and *rmtD*, and (3) sequencing of the amplified genes would have been important to distinguish *aac*(*6*′)-*Ib* from *aac*(*6*′)-*Ib*-*cr*. Hence, we suggest conducting parallel studies across different populations to expand the scope of research by investigating more aminoglycoside resistance mechanisms utilizing a larger number of pseudomonal isolates to improve global therapeutic outcomes. Furthermore, a future study is recommended to evaluate whether the isolates are XDR or DTR-*P. aeruginosa.*

## Figures and Tables

**Table 2:** DD and BM-MIC methods (μg/mL) of *P. aeruginosa* clinical isolates.

Antibiotic/Method	Disk Diffusion No. (%) of Isolates			Broth Microdilution MIC (µg/mL)										Resistant Rate (%)
	Resistant (R)	Intermediate (I)	Susceptible (S)	1024–256	128	64	32	16	8	4	2	1	0.5	
Gentamicin (GM)	29	-	71	4	-	3	4	18	2	7	6	6	50	31%
Amikacin (AK)	18	6	76	3	5	10	5	29	1	3	6	-	38	18%
Tobramycin (TM)	18	3	79	1	11	6	3	10	5	15	9	29	11	18%
Ceftazidime (CTZ)	9	10	81	2	2	1	5	15	26	6	7	14	22	10%
Imipenem (IMP)	29	-	71	2	1	1	7	21	7	28	7	6	20	32%
Piperacillin/Tazobactam * (TZP)	13	-	87	6	7	5	6	6	9	10	10	21	20	13%
Ciprofloxacin (CIP)	27	-	73	-	-	-	1	2	6	20	1	18	52	29%

Notes: The breakpoints were employed according to CLSI. When available, susceptible, intermediate, and resistance breakpoints are indicated by different colors (red for resistant, orange for intermediate, and yellow for susceptible. * = constant concentration of tazobactam = 4 µg/mL.

**Table 3:** ARGs profile of pan aminoglycoside isolates.

Isolate	*aph*(*3*)-*VI* (780 bp)	*Arm A* (846 bp)	*aac*(6*)-*Ib* (472 bp)	*aac*(6*)-*II* (542 bp)	*aac*(3*)-*II* (877 bp)	*rmtB* (769 bp)	*ant*(3**)-*I* (787 bp)
# 1	+	-	+	-	-	-	-
# 2	-	-	+	-	-	-	-
# 3	-	-	+	-	-	-	-
# 4	-	-	-	-	-	-	-
# 5	-	-	+	-	-	-	-
# 6	-	-	+	-	-	-	+
# 7	-	-	+	-	-	-	-
# 8	+	-	+	-	-	-	-
# 10	-	-	+	-	-	-	+
# 11	-	-	+	-	-	-	-
# 13	+	-	+	-	-	-	-
# 14	-	-	+	-	-	-	-
# 16	-	-	+	-	-	-	+
# 17	-	+	+	+	+	-	+
# 19	-	-	+	-	-	-	+
# 20	-	-	+	-	-	-	+
# 22	-	-	+	-	-	-	-
# 23	-	-	+	-	-	-	-

## Data Availability

Data is contained within the article or Supplementary Material.

## Supplementary Materials

The following supporting information can be downloaded at: https://www.mdpi.com/article/10.3390/microorganisms11092293/s1, Table S1: Correlation of each isolate with the clinical origin (different body parts at both male and female wards, including sputum (n = 44), urine (n = 36), blood (n = 7), wound swap (n = 7), bile (n = 2), and (n = 1) for each of eye swab, vaginal swab, peritoneal fluid, and catheter tip), Table S2: Paired *t*-test statistical analysis correlation among both the DD and the BM-MIC methods in means of (A) Hypothesis Test for the difference of two means: dependent sample (Paired *t*-test) − % (R/S) [percentage of resistant/susceptible isolates], and (B) Hypothesis Test for the difference of two means: dependent sample (Paired *t*-test) − % (R + I)/S [total number of resistant and intermediate isolates/susceptible isolates]. Figure S1: *GyrA* PCR amplification and sequence alignment, (A) *GyrA* PCR amplification of samples (1G–14G), 3 µL per Lane showed the band at Mwt of 365 bp, utilizing 100 bp ladder, (B) *GyrA* nucleotide sequence alignment versus the reference sequence from the GenBank nucleotide sequence database with accession numbers L29147.

## Abbreviations

Aminoglycoside modifying enzymes (AMEs), disk diffusion method (DD), broth-microdilution MIC testing (BM-MIC), antimicrobial resistance (AMR), aminoglycosides resistant genes (ARGs), multiple drug resistance (MDR), the Clinical and Laboratory Standards Institute (CLSI), National Health-care Safety Network (NHSN), Extensively-Drug Resistance (XDR) and Difficult-to-Treat Resistance (DTR).
