# Supplementary material for: Prevalence of Aminoglycoside Resistance Genes in Clinical Isolates of Pseudomonas aeruginosa from Taif, Saudi Arabia—An Emergence Indicative Study
Source: Microorganisms. 2023 Sep 12;11(9):2293. doi: 10.3390/microorganisms11092293 (PMC10537265; doi:10.3390/microorganisms11092293)
Supplement: Supplementary file 1 [file microorganisms-11-02293-s001.zip › microorganisms-2527209-supplementary.pdf]

microorganisms

MDPI

Table S1: Correlation of each isolate with the clinical origin (different body parts at both male and female wards, including sputum (n=44), urine (n=36), blood (n=7), wound swap (n=7), bile (n=2), and (n=1) for each of eye swab, vaginal swab, peritoneal fluid, and catheter tip).

P. aeruginosa ID	Ward	Source
#1	Male	sputum
#2	Male	sputum
#3	Male	sputum
#4	Male	sputum
#5	Male	sputum
#6	Male	sputum
#7	Male	sputum
#8	Male	sputum
#9	Male	sputum
#10	Male	sputum
#11	Male	sputum
#12	Male	sputum
#13	Male	sputum
#14	Male	sputum
#15	Male	sputum
#16	Male	sputum
#17	Male	sputum
#18	Male	sputum
#19	Male	sputum
#20	Female	sputum
#21	Female	sputum
#22	Female	sputum
#23	Female	sputum
#24	Female	sputum
#25	Female	sputum
#26	Female	sputum
#27	Female	sputum
#28	Female	sputum
#29	Male	sputum
#30	Female	sputum
#31	Female	sputum

#32	Female	sputum
#33	Female	sputum
#34	Female	sputum
#35	Male	sputum
#36	Male	urine
#37	Male	urine
#38	Male	urine
#39	Male	urine
#40	Male	urine
#41	Male	urine
#42	Male	urine
#43	Male	urine
#44	Male	urine
#45	Male	urine
#46	Male	urine
#47	Male	urine
#48	Female	urine
#49	Female	urine
#50	Female	urine
#51	Female	urine
#52	Female	urine
#53	Female	urine
#54	Female	urine
#55	Female	urine
#56	Female	urine
#57	Female	urine
#58	Female	urine
#59	Female	urine
#60	Female	urine
#61	Female	urine
#62	Female	urine
#63	Female	urine
#64	Female	urine
#65	Female	urine
#66	Female	urine
#67	Female	urine
#68	Male	blood
#69	Male	blood
#70	Male	blood
#71	Male	blood
#72	Female	blood

#73	Female	blood
#74	Female	wound swab
#75	Female	wound swab
#76	Female	wound swab
#77	Male	wound swab
#78	Male	eye swab
#79	Female	vaginal swab
#80	Male	peritoneal fluid
#81	Male	catheter tip
#82	Female	urine
#83	Female	sputum
#84	Female	sputum
#85	Male	urine
#86	Male	sputum
#87	male	bile
#88	Female	bile
#89	Male	sputum
#90	Male	sputum
#91	Female	sputum
#92	Male	blood
#93	Male	urine
#94	Male	sputum
#95	Female	wound swab
#96	Male	sputum
#97	Male	wound swab
#98	Female	wound swab
#99	Male	sputum
#100	Male	urine

Table S2. Paired *t*-test statistical analysis correlation among both the DD and the BM-MIC methods in means of (A) Hypothesis Test for the difference of two means: dependent sample (Paired *t*-test)- % (R/S) [percentage of resistant /susceptible isolates] and (B) Hypothesis Test for the difference of two means: dependent sample (Paired *t*-test)- % (R+I)/S [total number of resistant and intermediate isolates/susceptible isolates].

**A)**	**Hypothesis Test for the difference of two means: dependent sample (Paired t-test)- % (R/S)**						
	**Two-tailed test**	**Right tailed test**	**Left tailed test**	**Use this area to get x̄d and Sd**			
	**Ho: Ud not equal 0.0**	**≤ HO: Ud 0.0**	**≥ HO: Ud 0.0**	**DD % (R/S)**	**BM-MIC % (R/S)**	**difference**	**x̄d**
	Ha: Ud 0.00	Ha: Ud > 0.0	Ha: Ud < 0.0	0.4085	0.4203	-0.01183915	-0.043118
**Alpha level: a**	0.05	0.05	0.05	0.2368	0.2338	0.00307587	Sd
**Claimed difference: m**				0.2278	0.2278	0	0.06710999
**Mean differences: x̄d**	-0.0274	-0.0274	-0.0274	0.1111	0.1333	-0.02222222	N
**Standard deviation: S**	0.0426	0.0426	0.0426	0.4085	0.5246	-0.11613946	14
**Sample size: n**	7	7	7	0.1494	0.1494	0	
**Test Statistic**	-1.7014	-1.7014	-1.7014	0.3699	0.4143	-0.0444227	
**Critical Value**	±2.4469	1.9432	-1.9432				
**P-Value**	0.139760	0.9301	0.0699				
**Decision**	Do Not Reject Ho	Do Not Reject Ho	Do Not Reject Ho				
**Confidence Level**	0.95	0.05					

B)	**Hypothesis Test for the difference of two means: dependent sample (Paired t-test)- % (R+I)/S C)**						
	**Two-tailed test**	**Right tailed test**	**Left tailed test**	**Use this area to get x̄d and Sd**			
	**Ho: Ud = 0.0**	**≤ HO:Ud 0.0**	**≥HO: Ud 0.0**	**DD % (R+I)/S**	**BM-MIC% (R+I)/S**	**difference**	**x̄d**
	Ha: Ud not equal 0.00	Ha: Ud > 0.0	Ha: Ud < 0.0	0.4085	0.4493	-0.04082466	-0.058872
**Alpha level: a**	0.05	0.05	0.05	0.3158	0.2987	0.01708817	Sd
**Claimed difference: m**				0.2658	0.2658	0	0.08583939
**Mean differences: x̄d**	-0.0589	-0.0589	-0.0589	0.2346	0.3333	-0.09876543	N
**Standard deviation: S**	0.0858	0.0858	0.0858	0.4085	0.6393	-0.23089356	7
**Sample size: n**	7	7	7	0.1494	0.1494	0	
**Test Statistic**	-1.8146	-1.8146	-1.8146	0.3699	0.4286	-0.05870841	
**Critical Value**	±2.4469	1.9432	-1.9432				
**P-Value**	0.119517851482	0.9402	0.0598				
**Decision**	Do Not Reject Ho	Do Not Reject Ho	Do Not Reject Ho				
**Confidence Level**	0.95	0.05					

Note: Susceptible (S), Intermediate (I), and Resistant (R).

A)

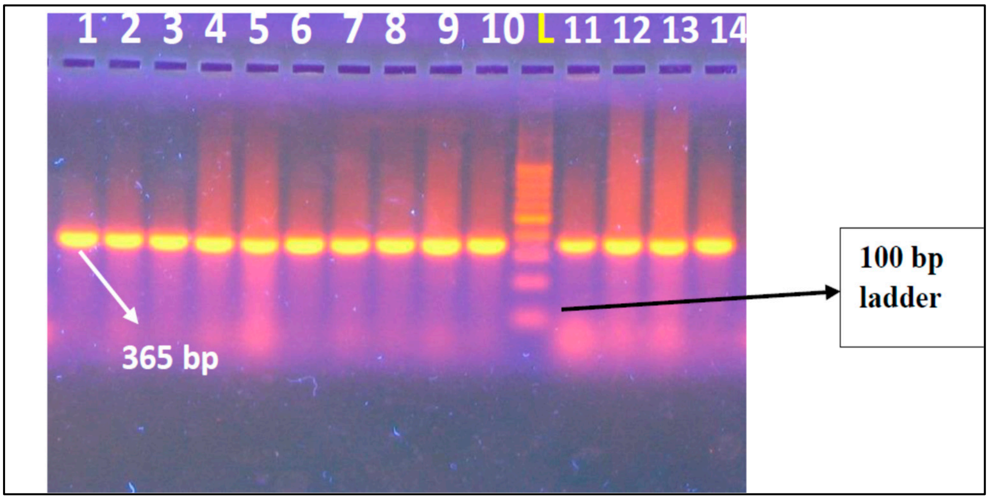

B)

```
Score            Expect     Identities        Gaps        Strand
544 bits(294)    2e-157     296/297(99%)      0/297(0%)   Plus/Plus

Query  468   TGTGGTCGGCGACGTGATCGGTAAGTACCACCCGCACGGCGACACCGCGGTCTACGACAC  527
             |||||||||||||||||||||||||||||||||||||||||||| |||||||||||||||
Sbjct  1     TGTGGTCGGCGACGTGATCGGTAAGTACCACCCGCACGGCGACATCGCGGTCTACGACAC  60

Query  528   CATCGTGCGCATGGCGCAGCCGTTCTCGCTGCGCTACATGCTGGTAGACGGCCAGGGCAA  587
             ||||||||||||||||||||||||||||||||||||||||||||||||||||||||||||
Sbjct  61    CATCGTGCGCATGGCGCAGCCGTTCTCGCTGCGCTACATGCTGGTAGACGGCCAGGGCAA  120

Query  588   CTTCGGTTCGGTGGACGGCGACAACGCCGCAGCCATGCGATACACCGAAGTGCGCATGGC  647
             ||||||||||||||||||||||||||||||||||||||||||||||||||||||||||||
Sbjct  121   CTTCGGTTCGGTGGACGGCGACAACGCCGCAGCCATGCGATACACCGAAGTGCGCATGGC  180

Query  648   CAAGCTGGCCCACGAACTGCTGGCGGACCTGGAAAAGGAAACCGTCGACTGGGTGCCCAA  707
             ||||||||||||||||||||||||||||||||||||||||||||||||||||||||||||
Sbjct  181   CAAGCTGGCCCACGAACTGCTGGCGGACCTGGAAAAGGAAACCGTCGACTGGGTGCCCAA  240

Query  708   CTACGATGGCACCGAGCAGATCCCGGCGGTCATGCCGACCAAGATTCCCAACCTGCT  764
             |||||||||||||||||||||||||||||||||||||||||||||||||||||||||
Sbjct  241   CTACGATGGCACCGAGCAGATCCCGGCGGTCATGCCGACCAAGATTCCCAACCTGCT  297
```

Figure S1: *GyrA* PCR amplification and sequence alignment, (A) *GyrA* PCR amplification of samples (1G-14G), 3µl per Lane showed the band at Mwt of 365 bp, utilizing 100 bp ladder, (B) *GyrA* nucleotide sequence alignment versus the reference sequence from the GenBank nucleotide sequence database with accession numbers L29147.

Disclaimer/Publisher's Note: The statements, opinions and data contained in all publications are solely those of the individual author(s) and contributor(s) and not of MDPI and/or the editor(s). MDPI and/or the editor(s) disclaim responsibility for any injury to people or property resulting from any ideas, methods, instructions or products referred to in the content.
